# Fast Homeostatic Plasticity of Inhibition via Activity-Dependent Vesicular Filling

**DOI:** 10.1371/journal.pone.0002979

**Published:** 2008-08-20

**Authors:** Kristin Hartmann, Claus Bruehl, Tatyana Golovko, Andreas Draguhn

**Affiliations:** Department of Physiology and Pathophysiology, University of Heidelberg, Heidelberg, Germany; Salk Institute for Biological Studies, United States of America

## Abstract

Synaptic activity in the central nervous system undergoes rapid state-dependent changes, requiring constant adaptation of the homeostasis between excitation and inhibition. The underlying mechanisms are, however, largely unclear. Chronic changes in network activity result in enhanced production of the inhibitory transmitter GABA, indicating that presynaptic GABA content is a variable parameter for homeostatic plasticity. Here we tested whether such changes in inhibitory transmitter content do also occur at the fast time scale required to ensure inhibition-excitation-homeostasis in dynamic cortical networks. We found that intense stimulation of afferent fibers in the CA1 region of mouse hippocampal slices yielded a rapid and lasting increase in quantal size of miniature inhibitory postsynaptic currents. This potentiation was mediated by the uptake of GABA and glutamate into presynaptic endings of inhibitory interneurons (the latter serving as precursor for the synthesis of GABA). Thus, enhanced release of inhibitory and excitatory transmitters from active networks leads to enhanced presynaptic GABA content. Thereby, inhibitory efficacy follows local neuronal activity, constituting a negative feedback loop and providing a mechanism for rapid homeostatic scaling in cortical circuits.

## Introduction

For proper information processing, the overall level of activity in neuronal networks has to be maintained within a physiological range between total silence and over-excitation, as it occurs within epileptic seizures. This functional homeostasis is reflected in network architecture which typically includes negative feedback by inhibitory interneurons, limiting the activity of excitatory neurons. However, the functional state of most central neuronal networks is highly dynamic, causing rapid changes in neuronal firing patterns, overall activity, and synchrony [1]. At the same time, associative memory mechanisms induce activity-dependent changes in synaptic weights. Therefore, homeostatic mechanisms must exist which adapt the balance between excitation and inhibition to the rapidly changing activity in the networks [2]. Indeed, homeostatic plasticity has been demonstrated at the level of intrinsic neuronal properties [3] and synaptic strength [4], [5]. The underlying mechanisms are, however, far less understood than classical Hebbian forms of plasticity.

Following prolonged periods of altered network activity, interneurons can alter the amount of GABA which is released for inhibitory synaptic transmission. For example, increased expression of the GABA-synthesizing enzyme glutamate decarboxylase (GAD) has been found in the hippocampus of chronically epileptic rats [6]. Conversely, GABA is down-regulated in deafferentiated somatosensory projection areas [7]. Under physiological conditions, one isoform of GAD (GAD65) is involved in defining critical period plasticity in the visual cortex [8]. Together, these findings suggest that the production of GABA contributes to homeostatic plasticity in neuronal networks. Consistent with this idea, recent evidence shows that changes in presynaptic GABA content do indeed alter inhibitory efficacy [4], [9]–[11]. It is unclear, however, whether this mechanism is also operant upon rapid changes of activity as present in dynamically changing networks. A possible mechanism for such activity-dependent, rapid adaptation is uptake of transmitters from the extracellular space. Axon terminals of inhibitory interneurons are equipped with membrane-bound uptake systems for GABA [12] and for glutamate [13], [14]. GABA can then be directly used for refilling of vesicles, while Glutamat is converted into GABA within the presynaptic bouton [15]–[17].

We hypothesized that uptake-mediated changes in presynaptic transmitter content can account for homeostatic scaling of GABAergic transmission upon rapid changes in network activity. This hypothesis requires i) that enhanced synaptic activity does enhance extracellular levels of GABA and glutamate; ii) that these transmitter molecules are rapidly and efficiently taken up by axon terminals of interneurons; iii) that the resulting cytosolic increase in GABA is sufficient to enhance filling of vesicles; and iv) that vesicles with enhanced GABA content cause significantly larger postsynaptic currents. In the present study tetanic stimulation of afferent fibers in the CA1 region of mouse hippocampal slices yielded a rapid and lasting increase in quantal size of miniature inhibitory postsynaptic currents. This potentiation is mediated by the release of larger quanta of GABA and can be blocked by combined inhibition of glutamate- and GABA-uptake. Thus, transmitters can be taken up from the extracellular space in an activity-dependent manner and are used to boost inhibitory synaptic efficacy. These findings provide a mechanism for rapid homeostatic scaling in cortical circuits, constituting a negative feedback loop between network activity and inhibitory synaptic efficacy.

## Results

### Up-scaling of GABAergic transmission following enhanced synaptic activity

We tested the idea that hippocampal interneurons homeostatically scale the strength of GABA_A_-mediated synaptic transmission as a function of local synaptic activity. We therefore applied high-frequency electrical stimulation to stratum radiatum of mouse hippocampal slices and recorded inhibitory synaptic activity from CA1 pyramidal cells in whole-cell configuration (see methods). Ionotropic glutamate receptors were blocked throughout in order to isolate GABAergic postsynaptic currents (IPSCs) and to avoid classical glutamate-mediated mechanisms of plasticity. Changes in inhibitory efficacy were measured at the level of single presynaptic vesicles by recording miniature IPSCs (mIPSCs) in the presence of TTX.

**Figure 1:**
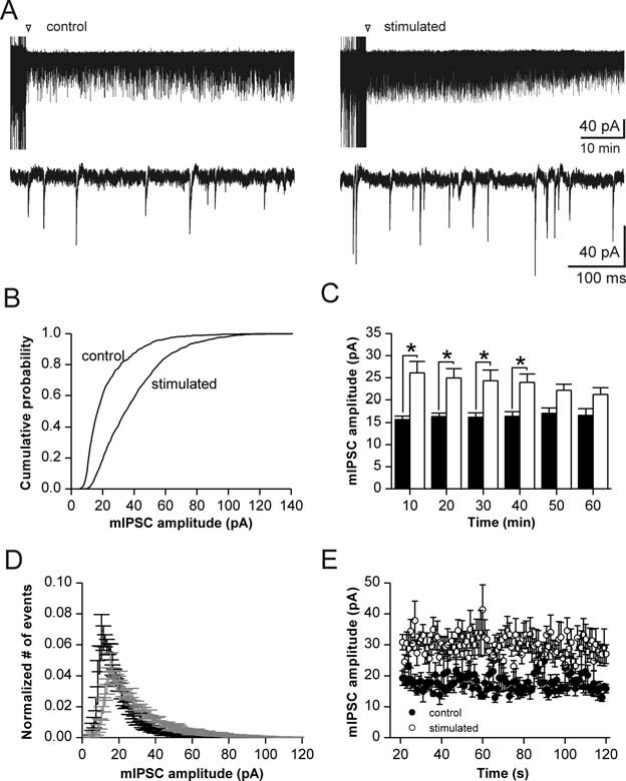
Increased miniature IPSC amplitudes following afferent fiber stimulation. (A) Original recordings of mIPSCs without (left) and with (right) afferent stimulation at 100 Hz for 1 s. Arrowheads indicate wash in of TTX and time of stimulation. Bottom traces show enlarged sections from long-lasting recordings illustrated above. Note apparent increase of amplitudes following stimulation. (B) Cumulative amplitude histograms from the experiments shown in A (data taken from the first 10 min after stimulation). Note shift towards larger amplitudes after stimulation (p<0.05; Kolmogorov-Smirnov test). (C) Median amplitudes of mIPSCs in cells from non-stimulated (black) and stimulated (open bars) slices for the recording period of 60 minutes. Significant increase of median amplitudes at 0 to 40 minutes after stimulation (n = 7 cells (control) and 9 cells (stimulated); p<0.05). (D) Normalized amplitude distribution of mIPSCs from control cells (black line) and stimulated cells (grey line). Note increased relative frequency of events with amplitudes >30 pA following stimulation. (E) Analysis of the first 100 s of recording after block of action potentials (20 s after stimulation). Open circles indicate median mIPSC amplitudes following stimulation which are increased from the earliest intervals analyzed (p<0.001, Wilcoxon matched pairs).

**Figure 2:**
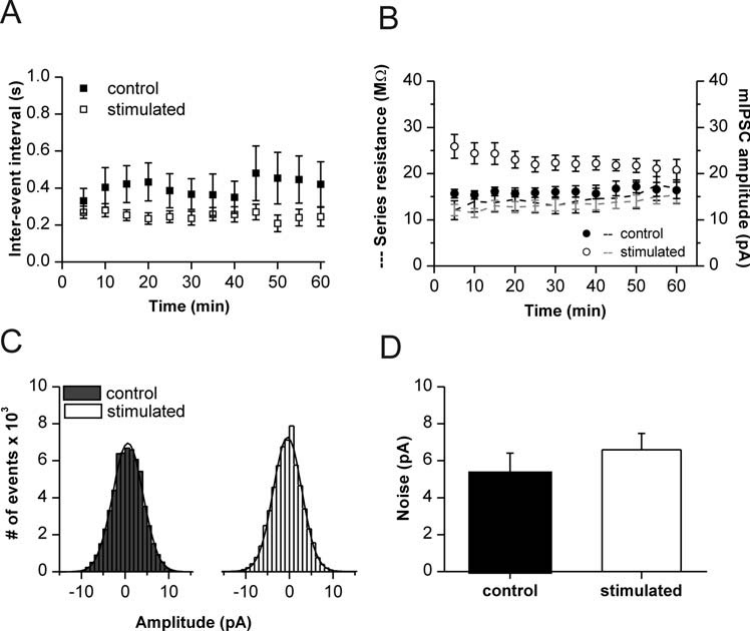
Stability of recording conditions. (A) Median inter-event intervals of mIPSCs in control (filled circles) and stimulated cells (open circles). Apparent decrease in intervals does not reach significance. (B) Median mIPSC amplitudes from control and stimulated cells (filled and open circles, respectively) are not correlated with series resistance during the recording period of 1 h (black line: mean R_s_ in control cells; grey line: mean R_s_ in stimulated cells). Values averaged for 5-min intervals. (C) Gaussian distribution of membrane noise from two representative cells (half-width 6.9 pA control and 6.5 pA stimulated). All-point histograms taken from 3 s of event-free raw data. (D) Mean baseline noise is similar for control cells and cells following afferent stimulation (5.3±1.1 pA, n = 6 versus 6.6±0.9 pA, n = 8).

**Figure 3:**
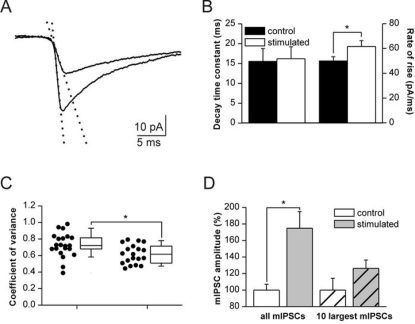
Properties of potentiated mIPSCs. (A) Averaged waveform of control and post-stimulation mIPSCs, taken from two representative example cells. Note steeper onset of the events in stimulated cell (dotted lines show linear fits to the 20–80% portion of the rising phase). (B) Decay time constants (left bars, mean±s.e.m.) are unchanged in stimulated cells while rate of rise (right bars) is significantly increased. (C) Coefficient of variance of mIPSC amplitudes is decreased after stimulation. Data taken from the first 10 min interval after application of TTX; n = 22 unstimulated cells and n = 19 cells after afferent stimulation. Boxes show mean value, 25 and 75% confidence intervals, and s.e.m., respectively. (D) Potentiation depends on quantal size. Left bars show the ∼70% increase in median amplitude when all detected mIPSCs are counted within the first 10 min after washin of TTX (p<0.05). Right bars (hatched) show the median amplitudes of the 10 largest mIPSCs from each cell. The apparent amplitude increase by ∼26% is not significant.

In CA1 pyramidal cells from unstimulated control slices we found a broad distribution of mIPSC amplitudes with a median quantal amplitude of 15.7±0.8 pA at −70 mV (n = 7; based on 1890±291 events/cell within the 10 min interval of evaluation). Amplitude and frequency of mIPSCs remained stable within a recording period of ∼60 min (Fig. 1A; Fig. 2B). In stimulated slices, network activity was enhanced by afferent stimulation immediately before application of TTX and recording of mIPSCs. This procedure resulted in enlarged mIPSCs (Fig. 1A), as confirmed by a clear rightward shift of the cumulative amplitude probability plot (Fig. 1B). For quantification, we analyzed median amplitudes of mIPSC distributions from 7 control cells and 9 stimulated cells, yielding a potentiation by ∼70% (median amplitude during the first 10 min after stimulation: 26.1±2.6 pA; n = 9; based on 3155±830 events/cell within 10 min). This increase in unitary amplitudes lasted for about 40 minutes before losing significance (p<0.05, Fig. 1C). Closer examination of amplitude histograms revealed that there was a pronounced increase in the proportion of medium-sized mIPSCs, whereas the number of large events or their absolute amplitude were not significantly enhanced (Fig 1D; Fig. 3D). Onset of this form of synaptic plasticity was fast. A detailed analysis of the initial phase revealed that the effect was already fully established at ∼20 s after stimulation (Fig. 1E). Thus, unitary inhibitory strength adapts rapidly to enhanced synaptic activity in the CA1 network.

While afferent stimulation caused a fast and sustained increase in mIPSC amplitudes, event frequency was less affected. Inter-event intervals of mIPSCs appeared slightly reduced after afferent stimulation, but were not significantly altered (Fig. 2A). A complicating factor in the assessment of mIPSC frequency is the signal-to-noise ratio which may change following altered network activity, causing systematic errors in event counts. In our cells, baseline noise was not different between unstimulated and stimulated slices (5.3±1.1 pA unstimulated vs. 6.6±0.9 pA; Fig. 2C, D). Together with the unchanged input- and series resistance (Fig. 2B), this finding does largely exclude that differences in event detection underlie the observed effects. It also suggests that afferent stimulation does not cause a lasting increase in tonic inhibition. Thus, enhanced synaptic activity in CA1 causes an increase of quantal IPSC amplitudes in pyramidal cells without gross changes in event frequency.

### Presynaptic location of activity-induced changes

**Figure 4:**
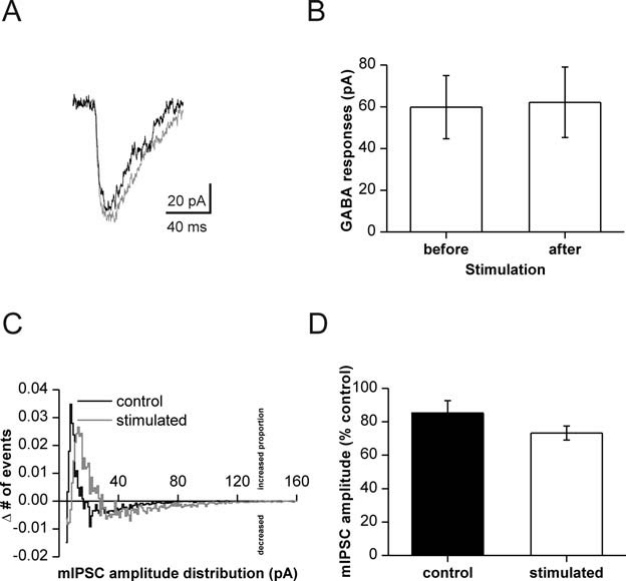
(A) Averaged example traces (n = 10) of GABA responses following UV-light induced uncaging of GABA. Black and grey lines show responses before and after afferent stimulation, respectively. (B) Similar amplitudes of GABA-induced currents before and after stimulation. Data from 9 cells, showing no significant difference in postsynaptic GABA sensitivity. (C) Differences in normalized amplitude distribution of mIPSCs induced by TPMPA (200 μM). Black line: control cells; grey line: potentiated cells. In both groups of cells, relative frequency of events with medium amplitude is increased, while small and large events are less frequent. This effect is different between stimulated and control cells, respectively (p<0.0001, Wilcoxon matched-pairs signed-ranks test). (D) Supression of median amplitude by TPMPA. No significant difference was observed between control (n = 6) and stimulated cells (n = 6), respectively.

Changes in quantal size are traditionally explained by changes in postsynaptic responsiveness. Recent evidence indicates, however, that presynaptic vesicular transmitter content can also account for changes of mIPSC amplitudes [9], [10]. We therefore sought to identify the primary site of adaptation. First, we analyzed the kinetics of postsynaptic currents. Decay time course could be well approximated by monoexponential fits and revealed similar time constants for cells from unstimulated and stimulated slices, respectively (15.54±3.22 ms vs. 16.22±2.99 ms; Fig 3A, B). In contrast, the rising phase of events was significantly steeper following afferent stimulation (61.7±4.6 pA/ms versus 49.9±3.4 pA/ms; p<0.05; Fig. 3B), consistent with larger amounts of GABA being released [18]. Second, we calculated the coefficient of variance of mIPSC amplitudes which was significantly reduced in cells which underwent afferent stimulation (control: 0.73±0.15; stimulated: 0.62±0.11; p<0.01; Fig. 3C). Thus, mIPSC amplitudes became more homogeneous, consistent with a more homogeneously filled population of presynaptic vesicles [19]. Third, we checked whether the potentiation reaches a ceiling level for large mIPSCs. Taking the 10 largest mIPSCs from each cell we found no significant potentiation by afferent stimulation, in contrast to the whole population of events (Fig. 3D). Thus, potentiation was restricted to vesicles and synaptic sites with sub-saturating amounts of GABA. Fourth, we checked directly for changes in the number or sensitivity of postsynaptic GABA_A_R. We therefore used laser-flash photolysis of CNB-caged GABA (20 μM) for rapid activation of postsynaptic GABA_A_R before and after stimulation, respectively. Flashing onto the somatic membrane reliably caused inward currents with rapid rise and slower decay (Fig. 4A). The resulting GABAergic currents were similar in both conditions (peak amplitude 59.8±15.2 pA prior to stimulation versus 62.2±16.9 pA after stimulation, respectively; n = 9; Fig. 4B). While this approach may induce currents with an enhanced contribution of extrasynaptic receptors, it supports the notion that the postsynaptic responsiveness of GABA_A_ receptors was unchanged.

We also tested for the effect of the weak GABA_A_ receptor antagonist TPMPA. This drug reduces IPSC amplitudes but has lower efficacy when saturation of postsynaptic GABA_A_ receptors is high [4], [11], [20]. In both groups of cells, addition of TPMPA (200 μM) reduced the number of detectable events to ∼50% of control (data not shown). TPMPA was applied to the bath at 10 min following stimulation or at the corresponding time in non-stimulated neurons. We then calculated the change in amplitude distribution induced by TPMPA in each cell (Fig. 4C). The resulting histograms show a clear shift towards medium-sized events, at the expense of very small and very large mIPSCs. Moreover, this effect was more pronounced in stimulated than in non-stimulated neurons as indicated by the rightward shift of event counts in Fig. 4C (p<0.0001, Wilcoxon matched-pairs signed-ranks test). Median mIPSC amplitudes, however, were not differentially affected by TPMPA (Fig. 4D).

Thus, increased synaptic activity potentiates mIPSCs without affecting events with very large amplitude. Together with the faster mIPSC rise time, differential effect of TPMPA and unchanged postsynaptic GABA-sensitivity these findings are best compatible with an increase in vesicular GABA content, yielding a more homogeneous distribution of GABAergic vesicles.

### Transmitter uptake as a source of vesicular filling

**Figure 5:**
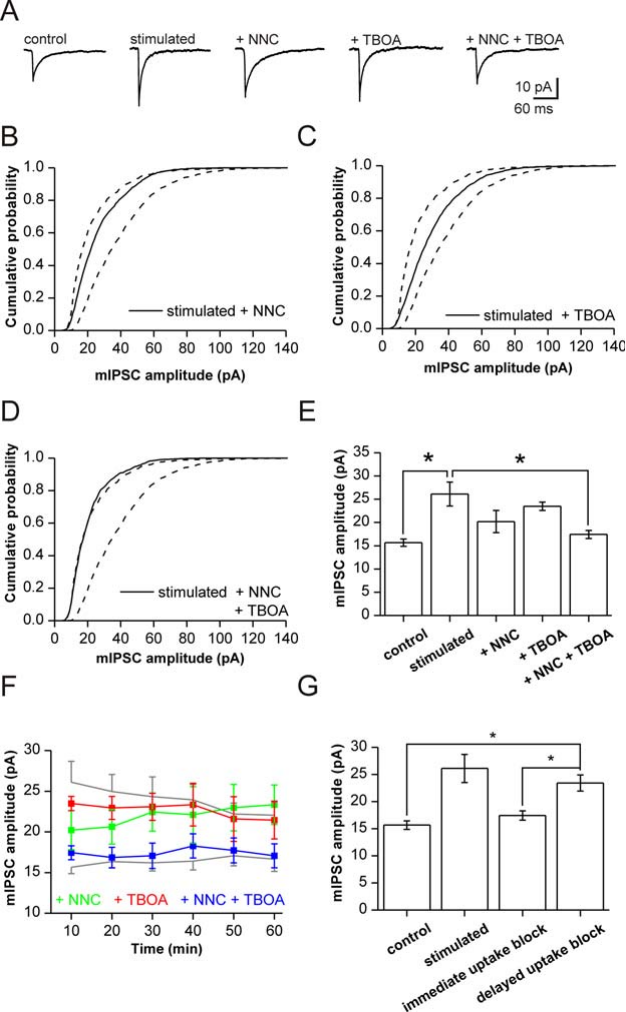
Prevention of mIPSC amplitude potentiation by blockers of GABA- and glutamate-uptake. (A) Averaged mIPSCs (n = 200 each) from representative cells recorded under control conditions; after stimulation; stimulation+NNC-711 (10 μM); stimulation+TBOA (100 μM); stimulation+NNC-711+TBOA. (B) Effect of NNC-711 (solid line) illustrated by cumulative amplitude distribution curves of the cells depicted in A. Curve is situated between distributions from control (left) and drug-free stimulated cell (right; dashed lines; data from Fig. 1A, B). (C) Effect of TBOA (solid line) for cell shown in A. Note shift towards larger amplitudes against control and smaller amplitudes against drug-free stimulated cell (dashed lines as in C). (D) Block of mIPSC potentiation in the presence of both uptake blockers (solid line). Note overlap of cumulative amplitude distribution with control data. (E) Summary of pharmacological experiments (mean±s.e.m. of relative median amplitude values, control cells = 100%). Numbers of cells: 7 control; 9 stimulated; 5 stimulated+NNC-711; 8 stimulated+TBOA; 7+NNC-711+TBOA. (F) Time course of median mIPSC amplitudes over 60 minutes of recording, analyzed in intervals of 10 min. Note the stability of control amplitudes (grey trace, bottom). Stimulation-induced amplitude increase decreases slowly over 60 min and is reduced by the application of NNC-711 (green trace) or by TBOA (red trace). Note complete loss of potentiation after combined application of NNC-711 and TBOA (blue trace). (G) Delayed application of uptake-blockers does not prevent or revert potentiation. Bar diagrams show means of median amplitudes. Note persistent potentiation after delayed application of NNC-711 and TBOA (at 10 min after establishing the activity-dependent potentiation).

In order to unravel the mechanisms underlying the increased quantal size, we sequentially blocked different sources of substrate supply for vesicular GABA filling. One major pathway is provided by the neuronal plasma membrane GABA transporter GAT-1 [21]. We therefore applied the GAT-1-selective GABA uptake blocker NNC-711 (10 μM) immediately after stimulation. As expected, the prolonged presence of GABA in the synaptic cleft slowed the decay time course of mIPSCs (Fig. 5A). NNC-711 reduced the stimulation-induced potentiation of mIPSC amplitudes. Median amplitude at 10 min post-stimulation was 20.2±2.4 pA as compared to 26.1±2.6 pA in drug-free potentiated slices and of 15.7±0.8 pA in control cells (Fig. 5E). While the apparent change in median mIPSC amplitude by NNC-711 was not significant, the amplitude distribution was clearly shifted to values between control and stimulated cells (Fig. 5B). Comparison of averaged cumulative amplitude distributions revealed that NNC-711 diminished the stimulus-induced potentiation (p<0.05; Kolmogorov-Smirnov test). The mIPSCs were, however, still larger than in non-stimulated controls (p<0.01, Kolmogorov-Smirnov test). Thus, the activity-dependent potentiation of mIPSCs depends partially, but not exclusively, on GABA uptake.

Recent evidence shows that uptake of glutamate can serve as an alternative source for vesicular GABA [10], [22]. Glutamate is taken up into presynaptic terminals of GABAergic neurons by the transporter EAAC1 [8], [9] where GABA can be synthesized by GAD65 [17]. Therefore, we repeated the experiment in the presence of TBOA (100 μM), a blocker of the neuronal plasma membrane glutamate transporter EAAC1. TBOA blunted the stimulation-induced rise in median mIPSC amplitude (at 10 min post-stimulation: 23.5±0.9 pA; Fig. 5 A, E). Similar to the effect of the GABA uptake-blocker, median values of mIPSC amplitudes recorded with TBOA were not significantly different from control cells or drug-free stimulated cells (Fig. 5 E). However, cumulative amplitude histograms were again located between those from controls and from stimulated cells (Fig. 5C). Similar to NNC-711, averaged cumulative amplitude distributions were significantly different from both control (p<0.001; Kolmogorov-Smirnov test) and stimulated cells (p<0.02; Kolmogorov-Smirnov test). These data demonstrate a contribution of glutamate uptake to the activity-dependent plasticity of inhibition. However, EAAC1 can not fully account for the potentiating effect of stimulation.

Consequently, we combined both drugs to block GABA- as well as glutamate-uptake immediately after stimulation. In this situation, activation of afferent fibers failed to induce an increase in quantal size of mIPSCs (Fig. 5 A,E; median at 10 min 17.4±0.9 pA for stimulated cells versus 15.7±0.8 pA for controls; significantly different from amplitudes in drug-free stimulated slices; p<0.05). Accordingly, the respective cumulative amplitude distribution plot overlaps with that from control experiments (Fig. 5D). On average, cumulative distributions in the presence of both uptake blockers were similar to those of control cells (p>0.1, Kolmogorov-Smirnov test) but different from stimulated cells (p<0.001; Kolmogorov-Smirnov test). Median amplitude changes in the absence and presence of GABA- and glutamate-uptake inhibitors are summarized in Figs. 5E and 5F.

These data show that membrane transporters for GABA and for glutamate are both critically involved in the observed potentiation. A parsimonious explanation for our findings is that afferent stimulation causes a transient increase in extracellular levels of GABA and glutamate which are rapidly taken up into presynaptic terminals of interneurons. Alternatively, it is feasible that stimulation directly increases the efficacy of GAT-1 and EAAC1 which would also lead to increased presynaptic transmitter content. We tested for this possibility by delayed application of NNC-711 and TBOA after establishing the initial mIPSC potentiation. This delayed block of transmitter uptake should de-potentiate mIPSCs if enhanced activity of the transporters was required throughout the maintenance of the effect. However, this was not found experimentally. In contrast to the application of uptake-blockers immediately after stimulation (Fig. 5E), delayed application of the drugs at 10 min after the stimulation did not reverse the initially established potentiation (23.4±1.5 pA versus 26.1±2.6 pA, with and without blockers, respectively; Fig. 5G). These data exclude that a lasting potentiation of the transmitter transporters themselves contributes to the potentiating effect of afferent stimulation. They are rather consistent with uptake of the transmitters immediately after the stimulus which increases extracellular concentrations of GABA and glutamate. Thereby, transmitter uptake by GAT-1 and EAAC1 provides a direct coupling between actual network activity and inhibitory synaptic strength. The persistent potentiation upon late application of NNC-711 and TBOA does also rule out that the block of potentiation by early application of uptake-inhibitors is caused by receptor desensitization through enhanced extracellular levels of GABA or glutamate.

An alternative explanation for an activity-dependent increase in mIPSC amplitude is uptake of glutamate by glia cells, conversion into glutamine and export into terminals of interneurons [11], [23], [24]. However, stimulation-induced potentiation did also occur in the presence of the system A transport blocker MeAIB (5 mM, applied 5 min prior to stimulation; median 23.2±0.6 pA, n = 3; p>0.1 towards stimulated drug-free cells; Kolmogorov-Smirnov test). These data suggest that glutamine uptake is not a major source of additional GABA in the present form of plasticity.

### Contribution of transmitter-uptake to basal transmission

**Figure 6:**
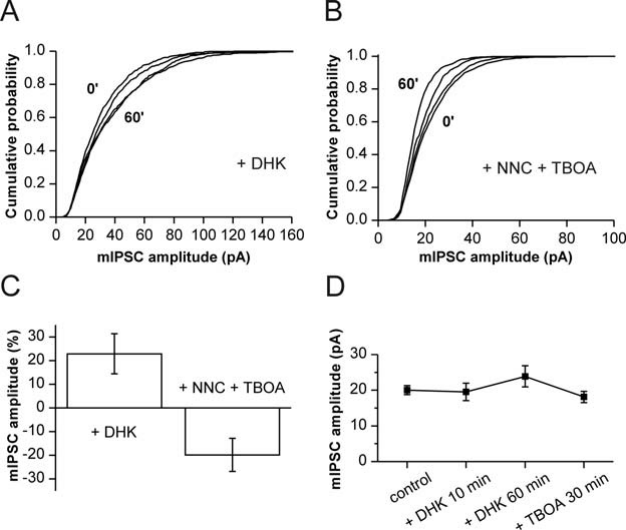
Alteration of miniature IPSC amplitudes without additional stimulation. (A) Successive mIPSC cumulative amplitude distributions at 0, 40, 50, 60 min from a cell in the presence of DHK (300 μM). Significant rightward shift after 40 min. (B) Successive mIPSC amplitude distributions in the presence of NNC-711 and TBOA. Significant leftward shift after 0, 40, 50, 60 min. (C) Changes in median amplitudes (mean±s.e.m.) of unstimulated cells after block of glial glutamate-uptake (left; 60 min in DHK; n = 4) and after block of neuronal GABA- and glutamate-uptake (right; 60 min in NNC-711+TBOA; n = 4). (D) Increase of median amplitude under DHK in two cells is reversed by addition of the neuronal glutamate uptake blocker TBOA.

As an independent test for the contribution of vesicular filling to the scaling of GABAergic synaptic strength we made use of the fact that most released glutamate is buffered by uptake into glia cells [25]. Suppression of this pathway should therefore divert glutamate from astrocytes to neurons, facilitating uptake of the GABA-precursor into inhibitory terminals. Application of the GLT-1 blocker dihydrokainate (DHK) without additional synaptic stimulation did indeed induce a continuous, slowly developing increase in mIPSC amplitude. Potentiation became significant after 30 min in 3/4 cells (Kolmogorov-Smirnov test; p<0.05) and reached 122.8±8.5% of control after 60 minutes (Fig. 6A, C; n = 4). This effect could be reversed by subsequent application of TBOA (Fig. 6D). This finding underlines the importance of neuronal glutamate uptake for GABAergic efficacy.

Finally, we reversed our experimental approach, trying to reduce vesicular GABA filling by blocking uptake of both transmitters. Simultaneous application of TBOA and NNC-711 reduced mIPSC amplitudes progressively over 60 minutes, reaching 80.2±7.0% of control (Fig. 6B, C; n = 4). The leftward shift of cumulative amplitude distributions became significant after 40 min in 3/4 cells (Kolmogorov-Smirnov test; p<0.05). Thus, uptake of transmitters from the extracellular space is crucial for the maintenance of inhibitory efficacy even under conditions of low synaptic activity in the hippocampal network.

## Discussion

### Homeostatic plasticity through regulation of transmitter content

**Figure 7:**
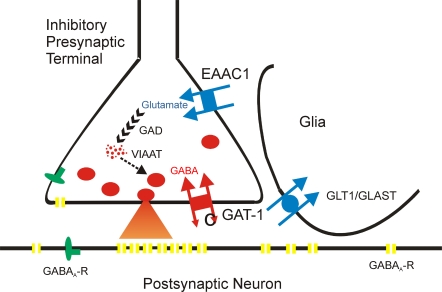
Schematic representation of the molecular scaling mechanism at inhibitory terminals. GABA is taken up through the neuronal GABA transporter GAT-1. In addition, glutamate is transported into the terminal by EAAC1. Subsequently, glutamate is converted into GABA by GAD65 which is directly coupled to the vesicular GABA transporter VIAAT. Both mechanisms modulate vesicular filling, depending on synaptic activity in the local environment. Glial glutamate transport is mediated by two different isoforms, GLT1 and GLAST. Reduction of glial glutamate uptake by the GLT1-blocker dihydrokainate (DHK) diverts glutamate into neuronal terminals, increasing vesicular GABA filling.

Our findings reveal that GABAergic synapses can rapidly adapt to changes in local network activity by changing vesicular transmitter content. Enhanced synaptic release of GABA and glutamate from endogenous stores is sufficient to boost the GABA content of inhibitory terminals. This effect is mediated by GABA- and glutamate-transporters which are both present at GABAergic synapses, allowing for fast adaptation of inhibition without a need for *de novo* protein synthesis or -transport (Fig. 7). Uptake-mediated plasticity provides a direct coupling between extracellular transmitter concentration and inhibitory synaptic strength. We show that the potentiation occurs upon a single tetanic stimulation of afferent fibers and exerts full efficacy within seconds. This mechanism constitutes a negative feedback loop which can stabilize excitation-inhibition-balance under conditions of dynamic network functions.

The potentiation of mIPSCs by uptake of previously released GABA and glutamate is likely to occur preferentially close to synapses which have been particularly active. There is a vast variety of GABAergic interneurons which project to defined laminar positions at CA1 pyramidal cells [26]. Thus, the effect will be most pronounced at specific sites of the target cells, providing an input-specific homeostasis of excitation and inhibition. In our somatic recordings, GABAergic mIPSCs were measured regardless of the site of the releasing terminal, probably leading to an underestimation of the local effect at the sites of release. Future analysis with recordings from pairs of pre- and postsynaptic cells or highly localized induction of transmitter release will be needed to resolve the role of defined subpopulations of interneurons for uptake-mediated homeostasis.

The stimulation protocol used in our approach is similar to conventional methods of LTP induction at Schaffer collateral synapses but was performed in the presence of blockers of ionotropic glutamate receptors. This rules out that the observed effects on synaptic inhibition are mediated by conventional NMDA-dependent plasticity at Schaffer collateral synapses. Additional effects by metabotropic glutamate receptors or by the release of further agents like serotonin, BDNF and others may well be present but cannot account for the immediate amplitude increase of miniature IPSCs which was fully blocked by combined inhibition of GABA- and glutamate-uptake, respectively.

Another confounding factor may be a change in release probability of GABAergic vesicles which may result in synchronous exocytosis of multiple vesicles from the same release site [27]. This effect is unlikely to underlie the present observations: there was no indication for any secondary peaks in quantal amplitude distributions, rise time of mIPSCs was shortened without indication for increased occurrence of events with multiple onset kinetics, and the potentiation was sensitive towards blockers of transmitter uptake which interfere with vesicular filling, rather than with release probability.

### GABA and glutamate transporters determine inhibitory strength

Most likely, GABA is taken up into the same terminal from which it has been released. Thereby, resting vesicles gain additional GABA from the content of previously released vesicles. GABAergic synapses in the hippocampus express GAT-1, a membrane-located high-affinity transporter of GABA [28]. A well-established function of the GABA transporter is to limit the duration of postsynaptic receptor activation at inhibitory synapses [29], [30]. However, it may also be essential for filling the presynaptic terminals with GABA, consistent with the epileptic phenotype of GAT-1-deficient mice [31] and with our present data. Blocking GABA uptake or suppressing the expression of GAT-1 has diverse cellular and systemic effects [32], [33], possibly due to the overlapping effects of reduced GABA supply, enhanced tonic inhibition [31], [34] and prolonged duration of inhibitory postsynaptic potentials [29]. Our present data confirm that GABA-uptake is an essential source for vesicular filling in active hippocampal circuits. Glutamate, on the other hand, can spill over from neighboring synapses [35], [36], providing a genuine addition to the transmitter pool. We found that part of the activity-dependent potentiation is blocked by TBOA, an inhibitor of the neuronal glutamate transporter EAAC1. Hippocampal interneurons express EAAC1 at their axon terminals [13], [14]. In stratum radiatum of CA1, these presynaptic terminals are localized in close vicinity to excitatory synapses [37]. Once taken up, glutamate is converted into GABA by GAD65, which resides in GABAergic terminals [15] and interacts directly with the vesicular GABA transporter VGAT [16], [17]. Consistent with this hypothesis, direct application of glutamate into hippocampal slices increases the amplitude of inhibitory postsynaptic currents [10]. Conversely, reduced expression of EAAC1 decreases IPSC amplitude and causes epileptic seizures [22]. Using stimulation-induced release of GABA and glutamate from endogenous stores, we have observed an activity-dependent increase in mIPSC amplitude which was fully blocked by combined inhibition of GAT-1 and EAAC1. Our findings thus suggest that a brief increase in transmitter release does supply sufficient GABA and glutamate to yield a rapid and lasting increase in quantal size of mIPSCs. This shows that the terminals of inhibitory neurons are highly sensitive and rapidly acting detectors for extracellular transmitters [38], [39], making presynaptic transmitter uptake a crucial element in homeostatic plasticity of hippocampal networks.

An alternative source for GABA is glutamine. While inhibitory axon terminals do not express appreciable amounts of glutamine-transporters and lack the glutamine-converting enzyme GLS1 [40], [41], recent evidence points towards a role for maintaining basal GABA levels at somato-dendritic sites [23], [24], or for activity-dependent regulation of vesicular GABA content by glutamine [11]. In our experimental approach, application of the system A transport inhibitor MeAIB [23] did not abolish the potentiating effect of afferent stimulation. Moreover, application of DHK is expected to reduce glial glutamate-glutamine conversion, probably resulting in lower glutamine-uptake by interneuron terminals. We found increased mIPSC amplitudes under these conditions, indicating that export of glutamine from glia cells is not a major limiting factor in our system. This does, however, not exclude a role for glutamine in the maintenance of basal GABA levels [23], [24] or in GABAergic transmission under conditions of increased activity [11].

### Activity-dependent vesicular filling determines quantal size

The observed increase in mIPSCs depends on two crucial prerequisites: first, that vesicles are not filled with saturating amounts of GABA, and, second, that non-potentiated vesicles are sub-saturating for postsynaptic GABA_A_ receptors. Although initial experimental and theoretical analysis of quantal inhibitory transmission in dentate granule cells revealed indication for postsynaptic saturation [42], several subsequent studies showed that unitary events of transmitter release are sub-saturating at inhibitory synapses in other regions, including CA1: It has been shown that the variable GABA content of presynaptic vesicles contributes to the variance of mIPSC amplitudes [19]. Increasing presynaptic GABA content by block of GABA-degradation does increase mIPSC amplitudes in CA3 pyramidal cells, supporting the view that vesicular GABA content and postsynaptic receptor occupancy are normally not saturated [9]. Similar findings have been made for glutamatergic synapses [43], neuromuscular junctions [44], and for dopaminergic synapses in midbrain neurons [45]. An independent proof for sub-saturating synaptic GABA concentrations at CA1 pyramidal neurons is provided by the potentiating effect of benzodiazepines at these synapses. Several observations show that these agents, which increase agonist affinity of GABA_A_ receptors, can potentiate the amplitude of miniature and evoked IPSCs. This effect is most parsimoniously explained by the additional activation of a receptor “reserve” [46], [47], although the major effect of benzodiazepines may be due to the prolongation of postsynaptic responses [48]. Similarly, increased uptake of glutamine results in increased amplitudes of evoked IPSCs in CA1, confirming the net increase in inhibitory efficacy upon enhanced vesicular GABA content [11].

Thus, inhibitory synapses in CA1 leave room for enhanced amplitudes after release of GABA-enriched vesicles. Recent experiments on inhibitory synapses with lowered GABA content found an increased efficacy of the weak competitive GABA_A_ receptor antagonist TPMPA [4], [11]. In our approach, GABA levels were enhanced, rather than decreased. Therefore, TPMPA induced different changes in amplitude distributions of control and stimulated cells, leaving more events with relatively large amplitudes in the latter. This finding is well compatible with increased transmitter content in a fraction of GABAergic vesicles following stimulation. The net effect on median mIPSC amplitude, however, was not different, probably reflecting the dilution of the effect by sampling from all (including non-potentiated) synapses and the confounding suppression of certain GABA_A_ receptor isoforms in CA1 pyramidal cells by TPMPA [49]. The finding that TPMPA induces a pronounced increase in medium-sized mIPSCs indicates that GABA content was preferentially enhanced in vesicles with low initial transmitter content. Possibly, highly loaded vesicles show saturation due to the decreasing electrochemical gradient at vesicular membranes [50]. Therefore, potentiation by enhanced transmitter release leads to a redistribution of vesicles, favoring those with medium, but sub-saturating GABA content.

### Non-Hebbian plasticity underlies network homeostasis

Rapid scaling of inhibitory synaptic efficacy is necessary for stable information transfer during Hebbian plasticity. In addition, multiple observations point towards non-Hebbian, homeostatic adaptations of inhibition during developmental [8], activity-dependent [3], [5] and pathophysiological plasticity [6], [7]. The present data show that excitation/inhibition-balance can be rapidly adjusted to the needs of the local network by linking the filling of GABAergic vesicles to acute changes in extracellular levels of synaptically released GABA and glutamate. Thereby, inhibitory quantal efficacy is coupled to local network activity, providing a fast negative feedback loop for homeostatic plasticity. This mechanism may also be relevant for the pathophysiology of epilepsy where prolonged periods of enhanced synaptic activity may increase vesicular GABA content, thereby contributing to spontaneous seizure arrest.

## Methods

### Hippocampal slice preparation

All animal procedures were approved by the state government of Baden-Württemberg and are in accordance with NIH guidelines. Experiments were performed on horizontal slices of the hippocampus (300 μm in thickness), prepared from young adult male C57Bl6 mice (>4 weeks) in ice cold sucrose solution which contained (in mM): sucrose 75; NaCl 87; KCl 2.5; NaH_2_PO_4_ 1.25; NaHCO_3_ 26; MgCl_2_ 7; CaCl_2_ 0.5; glucose 25, saturated with 95%O_2_-5%CO_2_. Subsequently, slices were incubated for 30 min in a submerged chamber at 34°C, followed by storage at RT in artificial cerebro-spinal fluid (aCSF) containing (in mM): NaCl 124; KCl 3; NaH_2_PO_4_ 1.25; NaHCO_3_ 26; MgSO_2_ 1.8; CaCl_2_ 1.6; glucose 10, saturated with 95%O_2_-5%CO_2_, pH 7.3 for 1–5 h before being placed in the submerged recording chamber.

### Whole cell recordings

Whole cell patch-clamp recordings were performed at RT on visually identified CA1 pyramidal neurons continuously superfused with aCSF. Pipette resistance was 2–5 MΩ, leading to series resistances of 8–16 MΩ which were controlled regularly every 5 min throughout the experiment. Currents were recorded in voltage clamp condition at −70 mV in symmetrical chloride (KCl-based intracellular solution; in mM: KCl 120; NaCl 5; CaCl_2_ 0.5; MgCl_2_ 2; HEPES 10; EGTA 5; pH 7.25; 285 mOsmol).

All drugs were obtained from Tocris, UK and applied via a rapid bath perfusion system. Recordings of mIPSCs were performed in continuous presence of APV 60 μM, CNQX 20 μM, CGP55845 1 μM, TTX 1 μM. GABA and glutamate transporters were blocked by NCC-711 10 μM, TBOA 100 μM and, DHK 300 μM as indicated in the respective results sections. MeAIB (α-(Methyl-amino) isobutyric acid was obtained from Sigma (Deisenhofen, Gemany) and added at 5 mM 5 min pior to stimulation or at the respective time point for control cells. O-(CNB-caged) GABA was obtained form Invitrogen, Germany and was used at a final concentration of 20 μM.

### Afferent Simulation

Increased network activity was simulated by afferent stimulation performed for 1s @ 100 Hz in the presence of glutamatergic and GABA_B_ receptor blockers. A bipolar tungsten electrode was placed upstream from the recorded cell at the inner border of the cell body layer, approximately halfway between stratum pyramidale and stratum radiatum. Initially, the strength of synaptic activation was assessed in current clamp condition. In these conditions, we applied consecutive stimuli with rising strength at intervals of 30 s. The effective stimulation strength was adjusted to yield action potentials in about 50% of trials in the postsynaptically recorded CA1 pyramidal neuron. We then switched to voltage clamp configuration and applied CNQX and APV to prevent conventional mechanisms of synaptic plasticity through ionotropic glutamate receptors. Immediately after the stimulus train, the bath solution was supplemented with 1 μM TTX which reliably suppressed action potentials within 20 s, enabling the isolation of mIPSCs.

### Laser photolysis

*O*-(CNB-caged) GABA, 20 μM was applied via bath perfusion. UV-light for flash photolysis was generated by a frequency tripled Nd:YAG laser (λ = 355 nm, 15 ns; Rapp OptoElectronic, Hamburg, Germany). Laser power was adjusted to 75% of its maximum. UV light was delivered to the objective plane of the microscope by an optical fiber and relayed through the 63× water immersion objective to form a spot of 2–3 μm. This spot was targeted to the soma of the recorded cell. The laser was gated to produce 15 ns long pulses which were used for photolysis of caged GABA (20 μM; 10 pulses delivered with 20 s intervals). GABA responses were stable during 10 subsequent applications at this interval.

### Data analysis

Currents were low-pass filtered at 3 kHz, digitally sampled at 20 kHz and analyzed offline (Spike/Signal, Cambridge Electronics Design, UK; OriginLabs, OriginLab Corporation, USA). mIPSC were identified by an automated event detection algorithm based on templates. Reliability of detection criteria was assessed by comparison with hand-evaluated individual traces. Effects on amplitudes are indicated by comparison of median values. Results from multiple cells are reported as mean±s.e.m. Statistical comparisons were based on non-parametric tests, using the Mann-Whitney-U test as not otherwise stated, a p-value<0.05 was considered as significant.
